# Pattern of Infectious Morbidity in HIV-Exposed Uninfected Infants and Children

**DOI:** 10.3389/fimmu.2016.00164

**Published:** 2016-05-06

**Authors:** Amy L. Slogrove, Tessa Goetghebuer, Mark F. Cotton, Joel Singer, Julie A. Bettinger

**Affiliations:** ^1^Division of Paediatric Infectious Diseases, Department of Paediatrics and Child Health, Faculty of Medicine and Health Sciences, Stellenbosch University, Tygerberg, South Africa; ^2^School of Population and Public Health, University of British Columbia, Vancouver, BC, Canada; ^3^Department of Paediatrics, St Pierre University Hospital, Brussels, Belgium; ^4^Université Libre de Bruxelles, Brussels, Belgium; ^5^Division of Pediatric Infectious Diseases, Department of Pediatrics, Vaccine Evaluation Center, BC Children’s Hospital, University of British Columbia, Vancouver, BC, Canada

**Keywords:** HIV exposed uninfected, HEU, infection, review, infant

## Abstract

**Background:**

Almost 30% of children in Southern Africa are HIV exposed but uninfected (HEU) and experience exposures that could increase vulnerability to infectious diseases compared to HIV unexposed (HU) children. The mechanisms of HEU infant vulnerability remain ill-defined. This review seeks to appraise the existing clinical evidence of the pattern of HEU infant infectious morbidity to aid understanding of the potential mechanism of susceptibility.

**Methods:**

A systematic search was conducted of scientific literature databases and conference proceedings up to December 2015 for studies comparing adequately defined HEU (in whom HIV-infection had been excluded through age-appropriate testing) and HU infants for all-cause mortality, all-cause hospitalization, or an infection-related morbidity. The systematic review was complemented by a narrative review of additional studies detailing the pattern of infectious morbidity experienced by HEU children without comparison to HU children or without conclusive exclusion of HIV-infection in HIV-exposed infants.

**Results:**

Only 3 of 22 eligible identified studies were designed to primarily compare HEU and HU infants for infectious morbidity. Fourteen were conducted prior to 2009 in the context of limited antiretroviral interventions. Three patterns emerge: (1) causes of morbidity and mortality in HEU infants are consistent with the common causes of childhood morbidity and mortality (pneumonia, diarrheal disease, and bacterial sepsis) but occur with greater severity in HEU infants resulting in higher mortality, more frequent hospitalization, and more severe manifestations of disease; (2) the greatest relative difference between HEU and HU infants in morbidity and mortality occurs beyond the neonatal period, during mid-infancy, having waned by the second year of life; and (3) HEU infants are at greater risk than HU infants for invasive streptococcal infections specifically Group B *Streptococcus* and *Streptococcus pneumonia*.

**Conclusion:**

To definitively understand HEU infant infectious morbidity risk, substantially larger prospective studies with appropriate HU infant comparison groups are necessary. HEU children would benefit from collaboration among researchers to achieve the quality of evidence required to improve HEU infant outcomes globally. HEU infant health and well-being, beyond avoiding HIV-infection, deserves a more prominent position in the local and international HIV research agendas.

## Introduction

Globally, approximately 1.4 million HIV-infected women become pregnant annually (1). Expanding vertical HIV transmission prevention (VTP) programs have markedly reduced HIV transmission, but for those HIV-exposed but uninfected (HEU) children, there are consequences of being born to an HIV-infected mother (1). HEU infants experience numerous exposures during fetal and early life that could increase their vulnerability to infectious diseases during infancy compared to HIV unexposed (HU) infants. Risk factors for HEU infant infectious morbidity and mortality can be divided into two groups: (1) Universal infant risk factors. These include prematurity or small for gestational age, absence of or suboptimal breastfeeding, maternal mortality, exposure to infectious agents particularly tuberculosis (TB), and poverty (2–11); (2) risk factors unique to HIV-exposed infants. These include exposure to the *in utero* environment altered by HIV, including exposure to viral proteins and glycoproteins, a pro-inflammatory state in the mother, maternal immune compromise, exposure to antiretroviral (ARV) and other drugs *in utero* and in breast milk (12–18). Understanding whether HIV-exposure plays a direct role in HEU infant risk for morbidity and mortality requires consideration of the universal infant risk factors as confounders to the relationship between HIV-exposure and infectious morbidity.

A number of perturbations of the HEU infant immune system, most likely as a result of HEU-unique exposures, have been observed (19). However, these immune system changes have not yet been studied in direct association with risk for infectious morbidity, probably the consequence of greatest importance for the majority of HEU infants and children living in regions experiencing high infectious disease-related child mortality (15, 20–26). Interrogation of the clinical pattern of infectious morbidity in HEU children could aid understanding of the potential mechanism of susceptibility and define a focus for further immunologic investigations within the vastness of the infant immune system. To this end, this review seeks to better understand the existing evidence of HEU infant vulnerability for infectious disease morbidity. Detailed reviews on HEU infant innate and humoral immunity are included in this special issue and will not be considered here.

## Method

This paper comprises a systematic review of the literature for studies comparing infectious morbidity in HEU and HU infants and children, as well as a narrative review of two additional types of studies, the first detailing the pattern of infectious morbidity experienced by HEU infants and children without comparison to HU children, and the second, without conclusive exclusion of HIV infection in HIV-exposed infants. For the systematic review, the literature was searched for studies comparing adequately defined HEU (in whom HIV-infection had been excluded through age-appropriate serologic or virologic testing) and HU infants for all-cause mortality, all-cause hospitalization, or an infection-related morbidity. Databases searched up to December 2015 included Medline, Embase, Cochrane Central Register of Controlled Trials, Cochrane Database of Systematic Reviews, and the following conference proceedings: International AIDS Society Conference on HIV Pathogenesis, Treatment and Prevention; International AIDS Conference; Conference of Retroviruses and Opportunistic Infections. Reference lists of identified manuscripts were scrutinized for additional studies. The search term strategy is detailed in Table 1. The systematic search identified 498 studies. Following title review, the abstracts of 149 studies and full-text of 32 studies were reviewed. Twenty-two eligible studies are summarized, and their evidence is discussed.

**Table 1:** **Search term strategy**.

	Search terms
1.	HIV infect* OR HIV OR HIV-1 OR HIV-2 OR HIV1 OR HIV2 OR human immune*
2.	Infant OR child OR infan* OR childr* OR paediatric OR pediatric
3.	Mortality OR morbidity OR infectio* OR diarrhoea OR diarrhea OR diarrh* OR pneumonia* OR pneumo* OR respiratory OR sepsis OR septic* OR bacteria* OR virus OR viral
4.	HIV exposed uninfected OR HEU OR HIV-EU OR HIV unexposed OR HUU OR HU OR HIV-UE OR HIV-uninfected
5.	1 AND 2 AND 3 AND 4

## Review

Most of what is known about HEU infant infectious morbidity comes from observations made early in the epidemic describing the natural history of HIV infected children compared to HIV-uninfected children, both HIV exposed and unexposed, or from secondary analyses of VTP clinical trials often without an HU control group (16, 27–32). Twenty-two studies comparing adequately defined HEU to HU infants for all-cause mortality, all-cause hospitalization, or infectious morbidity were identified, 21 from sub-Saharan Africa and 1 from Denmark (17, 27–29, 33–51). Among the 21 African studies, 14 were conducted prior to 2009 in the context of limited ARV interventions for either maternal health or VTP. Only three studies were designed to primarily compare these two groups of infants (Table 2 studies 11, 17, and 21) (41, 46, 50). A major limitation of these studies is either due to study design or measurement limitations, they have not consistently considered confounding by universal infant risk factors of infectious morbidity and mortality; therefore, the role of direct HIV-exposure on infectious disease susceptibility is unclear.

**Table 2:** **Studies comparing mortality and infectious morbidity in HIV-exposed uninfected and HIV-unexposed children in sub-Saharan Africa**.

Reference	Country (year)	Study type; sample size (HEU:HU)	Outcome	Follow-up	Findings [HEU vs. HU; point estimate (95% CI)]
**All-cause mortality and hospitalization**					
1. Spira et al. (28)	Rwanda (1989–1994)	Prospective cohort; (138:209)	Natural history of HIV in children	0–60 months	Severe pneumonia aHR 1.4 (0.9, 2.0); hospitalization aHR 1.3 (0.9, 1.4)
2. Taha et al. (29)	Malawi (1994–1997)	VTP RCT (499:119)	Morbidity	6–36 months	No difference in numerous morbidities
3. Brahmbhatt et al. (17)	Uganda (1994–1998)	Community surveillance (269:3183)	All-cause mortality	0–24 months	Mortality 18 months aIRR 1.16 (*p* < 0.05)
4. Marinda et al. (33); Koyanagi et al. (34)	Zimbabwe (1997–2000)	VTP RCT (3135:9210)	All-cause hospitalization and mortality	0–24 months	Mortality: 2–6 months aIRR 5.5 (3.9, 7.9); throughout 24 months aIRR >2 (1.2, 7.9); hospitalization: neonatal aIRR 1.5 (1.2, 2.0); post-neonatal aIRR 1.2 (0.9, 1.6)
5. Shapiro et al. (35)	Botswana (2000–2005)	VTP RCT (534:137)	All-cause hospitalization and mortality	0–24 months	Mortality aRR 4.2 (*p* < 0.01); Hospitalization aRR 2.17 (*p* < 0.001)
6. Homsy et al. (36)	Uganda (2007–2008)	Prolonged co-trimoxazole RCT (185:100)	Adverse event and hospital admissions	0–48 months	No difference in incidence of hospitalization or grade 3/4 adverse events
7. Landes et al. (37)	Malawi (2008–2009)	Retrospective VTP program evaluation (128:200)	All-cause hospitalization	0–20 months	No difference in hospitalization
8. Moraleda et al. (38)	Mozambique (2008–2009)	Prospective cohort (Total 153)	Sick clinic visits and hospitalization	0–12 months	Sick clinic visit: uIRR 0.79 (0.63, 0.99); Hospitalization: uIRR 1.51 (0.71, 3.18)
9. Slogrove et al. (39)	South Africa (2009–2011)	Prospective cohort (27:28)	Infectious cause hospitalization	0–12 months	Sick clinic visit: uIRR 1.06 (0.79, 1.39); hospitalization: uIRR 2.74 (0.75, 8.78)
10. Marquez et al. (40)	Uganda (2010–2013)	Malaria prevention RCT (186:389)	Morbidity and all-cause mortality	6–24 months	No difference breastfed HEU and breastfed HU 6–11 months old
11. Slogrove^c^ (41)	South Africa (2012–2014)	Birth cohort (94:82)	Infectious cause hospitalization	0–6 months	Sick clinic visit: uIRR 0.82 (0.58, 1.16); all hospitalization: aOR 1.45 (0.4, 4.5); very severe hospitalization: aOR 4.2 (1.0, 19.2)
**Diarrhea-specific morbidity**					
12. Thea et al. (27)	DRC (Zaire) (1989–1992)	Prospective cohort (139:191)	Diarrhea incidence	0–12 months	Persistent diarrhea incidence 4.9 vs. 2.7/100 py (*p* = 0.23)
13. Rollins et al. (42)	South Africa (2001–2005)	Breastfeeding intervention cohort (936:115)	Diarrheal morbidity and all-cause mortality	0–18 months	Diarrhea at 6 months aHR 1.45 (0.75, 2.79); mortality 12 months aHR 0.77 (0.49, 1.21);
14. Pavlinac et al. (43)	Kenya (2011–2013)	Cross-sectional diarrhea surveillance (105:821)	Enteric pathogen prevalence	6–180^b^ months	*Cryptosporidum* spp. aPR 2.7 (1.3–5.6)
**Pneumonia-specific morbidity**					
15. Izadnegahdar et al. (44)	South Africa (1999–2001)	Severe pneumonia RCT (40:244)	Severe pneumonia treatment failure	3–59^b^ months	uOR 2.19 (0.75–2.75)
16. McNally et al. (45)	South Africa (2001–2002)	Hospital cohort (41:75)	Severe pneumonia treatment failure	0–12^b^ months	aOR 6.02 (1.55, 23.38)
17. Kelly et al.^c^ (46)	Botswana (2012–2013)	Prospective hospital cohort (64:153)	Severe pneumonia treatment failure	1–23^b^ months	Treatment failure: aRR1.83 (1.27, 2.64); Mortality aRR 4.31 (1.44, 12.87)
18. Le Roux et al. (47)	South Africa (2012–2014)	Birth cohort (130:567)	Pneumonia incidence	0–12 months	All pneumonia: aIRR 1.62 (1.0, 2.6); severe pneumonia: uIRR 4.04 (1.5, 10.8)
**Other specific morbidity**					
19. Cutland et al. (48)	South Africa (2004–2007)	RCT (1255:5804)	Neonatal sepsis incidence	0–1 month	Propensity score matched analysis: early onset (20.6 vs. 33.7/1000 live births); late onset (5.8 vs. 4.1 per 1000 live births)
20. Madhi et al. (49)	South Africa (2009–2010)	Cross-sectional acute otitis surveillance (63:182)	Otopathogen prevalence	3–59^b^ months	No difference in type of otopathogens
21. Von Mollendorf et al.^c^ (50)	South Africa (2009–2013)	Laboratory surveillance (253 HEU cases:377 HU cases)	IPD incidence^a^ and mortality	0–12^b^ months	IPD aRR 0–6 months 3.6 (2.8, 4.6); mortality due to IPD aOR 1.76 (1.09, 2.85)

^a^Calculated using a population-based denominator.

^b^Age at time of event.

^c^Designed to primarily compare HEU and HU infants for an infection-related morbidity.

DRC, Democratic Republic of Congo; HEU, HIV exposed uninfected; HR, hazard ratio; HU, HIV unexposed; IPD, invasive pneumococcal disease; IRR, incidence rate ratio; OR, odds ratio; PR, prevalence ratio; RCT, randomized controlled trial; RR, risk ratio; VTP, vertical transmission prevention; prefix “a,” adjusted; prefix “u,” unadjusted.

### All-Cause Mortality and Hospitalization

Studies conducted early in the epidemic did not observe significant differences in mortality, hospitalization, or infectious morbidities between HEU and HU children (28, 29). The first study to definitively demonstrate a higher mortality and morbidity in HEU compared to HU infants was the Zimbabwe Vitamin A for Mothers and Babies Project (ZVITAMBO) (Table 2 study 4), detailed further in this issue (33, 34). This large cohort study was conducted prior to the availability of either ARVs for treatment or prevention of HIV transmission or cotrimoxazole chemoprophylaxis, and 94% of infants breastfed until 12 months of age. The study demonstrated an almost four times greater risk of mortality [incidence rate ratio (IRR) 3.9, 95% CI 3.2–4.8] at 12 months and two times greater risk of mortality (IRR 2.0, 95% CI 1.2–3.5) at 24 months in HEU compared to HU infants (33). Similar to HU infants, the highest absolute mortality for HEU infants was in the neonatal period. The relative increase in HEU mortality was greatest between 2 and 6 months of life, reaching 5.5 (95% CI 3.9–7.9) times that of HU infants (33). Eighty percent of HEU mortality occurred during the first 6 months of life. Morbidity, measured as sick clinic visits and hospitalizations, was also greater in HEU than in HU infants (34). Smaller Southern and Eastern African studies confirmed the elevated morbidity and mortality in HEU infants in this region (17, 35). The Mashi study in Botswana (Table 2 study 5) similar to ZVITAMBO observed an at least four times greater risk for mortality and double the risk for hospitalization at 6 and 24 months (35).

Two Ugandan studies designed primarily to evaluate prolonged co-trimoxazole preventive therapy for malaria chemoprophylaxis, or improvement of HEU health did not find substantial differences in infectious morbidity (Table 2 studies 6 and 10) (36, 40). A single study outside of sub-Saharan Africa, utilizing the Danish national registry compared 260 Danish HEU children to 1300 matched HU children for risk of hospital admission before age 4 years (51). The IRR for all hospitalizations was 3.2 (95% CI 2.8–3.7), but the difference between HEU and HU infants was in non-malignant hematological disease and other non-specific symptoms (IRR 3.2, 95% CI 1.5–7.0) rather than infectious disease hospitalizations (IRR 1.0, 95% CI 0.79–1.37). Three smaller Southern African studies, one from Mozambique and two from South Africa, showed an increased risk for hospitalization in HEU infants compared to HU infants while out-patient sick clinic visits remained similar in both groups (Table 2 studies 8, 9, and 11) (38, 39, 41). Vaccination uptake was similar in both HEU and HU infants in the South African studies (39, 41). One of the South African studies specifically considered the severity of hospitalization events and observed a four times greater probability of hospitalization for very severe infectious events in HEU compared to HU infants with comparable durations of breastfeeding (41). Inconsistencies in infectious morbidity outcomes between the Ugandan, Danish, and Southern African studies may reflect regional differences in infectious morbidity risk in HEU infants, differences in the age profile of children observed, with younger infants observed in South Africa and Mozambique, or alternatively, differences in measurement and analysis of infectious morbidity outcomes.

Cohorts in Europe and the Americas have reported rates of hospitalization and other morbidities in HEU children without comparison to HU control groups. The European Collaborative Study followed more than 1600 HEU infants from 1985 to 2002. A hospitalization rate of 264/1000 child-years was observed, and the probability of hospitalization was not associated with socio-demographic factors (52). In a cohort of infants born between 1985 and 2006 in Belgium, the incidence rate of severe infections was 16.8/100 HEU infant years (53). In Puerto Rico and the United States between 1989 and 2001, 24% (229/955) of HEU children were hospitalized in the first 2 years of life with respiratory tract infections occurring most frequently (16%) followed by gastroenteritis (11%) (54). A prospective study in Latin America and the Caribbean of HIV-infected mothers and their infants described infectious disease morbidity in 462 HEU infants up to six months of age between 2002 and 2004 (55). Hospitalizations occurred at least once in 17.5% of infants, the majority (53%) of admissions due to lower respiratory tract infections (LRTI). Although these studies have provided valuable descriptions of HEU infants, it is difficult to interpret these findings in the absence of comparison to HU infants.

Despite some inconsistencies, the existing evidence suggests that HEU infants experience greater mortality and hospitalization than HU infants. The risk ratios (RRs) for hospitalization from the most recent South African and Mozambican studies are approximately 1.5 times higher in HEU compared to HU infants and although imprecise, raise the possibility of a strong association between HIV exposure and infectious morbidity (38, 41). HIV exposure without HIV infection could account for up to one-third of infectious cause hospitalizations in HEU infants. In South Africa, for example, with an HIV exposure prevalence of almost 30% this would translate to 12.5% of infectious cause hospitalizations in all HIV-uninfected infants under 6 months of age occurring as a consequence of being born to an HIV-infected mother. Therefore, in a birth cohort of approximately one million in South Africa, an excess 35 000 infant hospitalizations would occur annually (41, 56, 57). Even a RR of 1.1 would produce an annual excess of 8100 infant hospitalizations in the same South African birth cohort, still a considerable number.

### Diarrheal Disease

One of the earliest observations of increased infectious morbidity in HEU infants was from a prospective cohort study in the Democratic Republic of Congo between 1989 and 1992 (Table 2 study 12) (27). HEU infants had almost double the incidence of persistent diarrhea than HU infants (4.9 vs. 2.7/100 person years) and the hazard of persistent diarrhea was increased further in HEU infants of mothers who died [hazard ratio (HR) 10.4, *p* < 0.005]. In a Kenyan study (Table 2 study 14), the prevalence of *Cryptosporidium* species was three times higher in HEU than HU children with acute diarrhea (43). The risk of diarrheal disease is intricately tied to infant feeding practices, presenting challenges in understanding whether diarrhea-related morbidity is due entirely to suboptimal breastfeeding in HEU infants or whether HIV exposure specific risk factors also play a role. In a South African non-randomized intervention cohort designed to determine the effect of exclusive breastfeeding on HIV transmission and to determine effectiveness of peer support on rates of exclusive breastfeeding (Table 2 study 13), all infants exclusively breastfed in the first 6 months of life presented with fewer diarrheal days than mixed or no breastfeeding with no difference between HEU and HU infants (42).

### Respiratory Infections

A number of studies have described the burden of respiratory tract infections in HEU infants, but without HU control groups, it has not been possible to understand whether these HEU infants are experiencing the morbidity expected of all infants in their context or something in excess of that (16, 54, 58, 59). In two studies with appropriate HU comparison groups, although the most common reason for admission of HEU infants was for a LRTI, this rate was no different than that in HU infants requiring hospitalization for LRTIs (Table 2 studies 5 and 11) (35, 41). In a third study, HEU infants had a marginally significantly greater risk than HU infants for all pneumonia (hospitalized and out-patient cases) in the first year of life. However, they were at substantially higher risk for severe pneumonia (Table 2 study 18) (47). Additionally, it has been consistently observed in South African and Botswanan studies that HEU infants fail empiric pneumonia treatment more often than HU infants (Table 2 studies 15–17) (44–46). Although HEU infants experience a substantial burden of respiratory tract infections as reflected by the incidence of disease, this incidence is no different to HU infants in the same settings (35, 47). These two groups of infants may differ in the severity of the LRTIs and this is an observation that deserves further evaluation.

### Bacterial Sepsis

Irrespective of HIV exposure, the risk for bacterial sepsis is highest during the neonatal period when the immature neonatal immune system is often unable to contain bacterial infections (60, 61). Considering all-cause neonatal sepsis in a large South African neonatal cohort, HEU neonates had a slightly lower rate than HU neonates for early onset (0–7 days of life) and similar rate for late onset (8–28 days of life) neonatal sepsis (Table 2 study 19) (48). This persisted in a second analysis using propensity score matching to deal with differences in potentially confounding variables between HIV-infected and HIV-uninfected mothers. Group B *Streptococcus* (GBS) infection though is significantly increased in Belgian and South African HIV-exposed neonates (62–64). A Belgian retrospective cohort study observed an almost 20 times (RR 19.7, 95% CI 7.5–51.7) greater risk of confirmed GBS infection in HEU infants compared to the population rate for HU infants between 2001 and 2008 (62). This increased risk was also associated with more severe manifestations of disease including septic shock, meningitis, and late-onset disease. In South Africa, HIV-exposed infants had a 70% greater risk of early onset GBS disease (IRR 1.69, 95% CI 1.28–2.24) and three times increased risk of late-onset GBS disease (IRR 3.18, 95% CI 2.34–4.36), with replication of these findings in a later study in the same population (63, 65).

A South African nationwide invasive pneumococcal disease (IPD) laboratory surveillance study showed that during the first 6 months of life HEU South African infants have a three times greater risk for IPD that includes pneumonia, bacterial septicemia, and meningitis when compared to HU infants, and almost double the probability of IPD mortality independent of infant pneumococcal vaccination (Table 2 study 21) (50). In keeping with this, a four times greater risk for IPD was observed in Belgian HEU infants, born between 1985 and 2006, compared to the general infant population prior to pneumococcal conjugate vaccination (53). The risk for all-cause bacterial sepsis has not been evaluated in HEU infants directly compared to HU infants beyond the neonatal period. The French Perinatal Cohort showed an association in HEU infants between serious bacterial infections and maternal CD4 count near delivery (66). In this study of 7638 infants, the adjusted HRs for a serious bacterial infection were 1.7 (95% CI 1.2–2.6) and 1.2 (95% CI 0.8–1.9) for infants of mothers with CD4 count below 350 cells/μl and 350–500 cells/μl respectively, compared to above 500 cells/μl. This suggests that severe maternal immune compromise may increase the risk for serious bacterial infections in HEU infants.

### Viral Infections

Vertical infection of HEU infants with other viral infections is important to consider, most notably due to cytomegalovirus (CMV), but also due to Human Herpes Virus 6 (HHV-6) (67, 68). Direct comparisons of rates of congenital CMV in HEU and HU infants have not been published. There is indirect evidence to suggest that HEU infants may experience greater risk of congenital viral infections. HIV-infected mothers more often transmit CMV and HHV-6 to their infants than HIV-uninfected mothers (68). In both the United States and South Africa, a congenital CMV prevalence of approximately 3% has been documented and is associated with features of advanced maternal HIV disease, high HIV viral load, and CD4 count of below 200 cells/μl (69, 70). The French Perinatal Cohort has observed a reduction in the prevalence of congenital CMV over time as combination ARV therapy has expanded, decreasing from 3.5% in 1997–1998 to 1.2% in 2003–2004 (67).

### Infectious Morbidity Pattern

From the studies reviewed, three patterns become evident:

(1) The causes of morbidity and mortality in HEU infants in sub-Saharan Africa are no different to the common causes of childhood morbidity and mortality in HU infants – pneumonia, diarrheal disease, and bacterial sepsis predominate. These common childhood infections may occur with greater severity in HEU infants resulting in a higher mortality, more frequent hospitalization, and more severe manifestations of disease (33–35, 38, 41, 45–47, 50);
(2) HEU infants are at greater risk than HU infants for invasive streptococcal infections specifically GBS and *Streptococcus pneumoniae* (50, 62, 63);
(3) The neonatal period presents an increased risk for infectious morbidity in all infants, irrespective of HIV exposure. The greatest relative difference between HEU and HU infants occurs beyond the neonatal period, during mid-infancy. This is evidenced by the absence of a difference in all-cause neonatal sepsis and the relative increase in mortality in HEU compared to HU infants peaking between 2 and 6 months of age (33, 48).

## Future Research Considerations

To definitively understand HEU infant infectious morbidity risk, substantially larger prospective studies with appropriate HU infant comparison groups are needed. HEU infants would benefit from enhanced collaboration among researchers to achieve the quality of evidence required to improve HEU infant outcomes globally. Agreement on standardized infectious morbidity outcome measurement would allow for collaboration of multiple cohorts while still pursuing unique individual hypotheses and objectives. It is fully recognized that enhanced efforts to improve uptake of sustained breastfeeding in HIV-exposed infants are required. However, there is evidence that supports that pathways other than deprivation of breast milk require consideration and study to comprehensively understand HEU infant risk for infectious morbidity (33, 34, 41, 71). In designing further studies and looking for possible interventions to secure HEU infant well-being, researchers need to be cognizant of the vulnerabilities that all infants and children experience, whether HIV exposed or not. Interventions that are found to improve health in HEU infants may very well also improve outcomes for HU infants. The bigger picture of improving health and well-being for all infants particularly in Africa needs to be kept in mind. HEU infants comprise almost one-third of the infant population in high burden countries and their health and well-being, beyond avoiding HIV-infection, deserve a more prominent position in the local and international HIV research agendas.

## Author Contributions

AS conceived of the review, conducted the systematic review, and wrote the first draft of the manuscript. MC, JS, and JB supervised the work of AS and provided editorial input into the manuscript. TG provided editorial input into the manuscript.

## Conflict of Interest Statement

The authors declare that the research was conducted in the absence of any commercial or financial relationships that could be construed as a potential conflict of interest.
